# Children's traditional ecological knowledge of wild food resources: a case study in a rural village in Northeast Thailand

**DOI:** 10.1186/1746-4269-3-33

**Published:** 2007-10-15

**Authors:** Chantita Setalaphruk, Lisa Leimar Price

**Affiliations:** 1 Social Sciences Department, Wageningen University and Research Centre, Wageningen, The Netherlands

## Abstract

Consuming wild foods is part of the food ways of people in many societies, including farming populations throughout the world. Knowledge of non-domesticated food resources is part of traditional and tacit ecological knowledge, and is largely transmitted through socialization within cultural and household contexts. The context of this study, a small village in Northeast Thailand, is one where the community has experienced changes due to the migration of the parental generation, with the children being left behind in the village to be raised by their grandparents.

A case study approach was used in order to gain holistic in-depth insight into children's traditional ecological knowledge as well as patterns of how children acquire their knowledge regarding wild food resources. Techniques used during field data collection are free-listing conducted with 30 village children and the use of a sub-sample of children for more in-depth research. For the sub-sample part of the study, wild food items consisted of a selection of 20 wild food species consisting of 10 species of plants and 10 species of animals. Semi-structured interviews with photo identification, informal interviews and participatory observation were utilized, and both theoretical and practical knowledge scored. The sub-sample covers eight households with boys and girls aged between 10–12 years old from both migrant families and non-migrant families. The knowledge of children was compared and the transmission process was observed.

The result of our study shows that there is no observable difference among children who are being raised by grandparents and those being raised by their parents, as there are different channels of knowledge transmission to be taken into consideration, particularly grandparents and peers. The basic ability (knowledge) for naming wild food species remains among village children. However, the practical in-depth knowledge, especially about wild food plants, shows some potential eroding.

## Background

In many locations around the world, as in rural Northeast Thailand, settled farmers have a reliance on wild food resources gathered from the agricultural landscape (fields, ditches, pathways) as well as from within the villages in which they reside [1-8]. Some of the plant foods in Northeast Thailand can be considered managed in that transplanting and protection are undertaken [9-11]. In addition, there are many small protein items collected including freshwater shrimp and crabs, frogs and insects. Hunting activities which include birds, rats, lizards and other small game and fishing are common [12,13].

The contributions of edible wild resources to the well-being of rural households are wildly recognized in many different respects. They are vital components to the daily diet and nutrient intake and are particularly important during times of crisis [8,12,14-19]. They can be found close to human settlements for ease of acquisition and provide nourishment and variety in the diet [20,21]. These edible wild resources can also contribute to health maintenance as functional or medicinal foods [22], as well as provide rural households with supplemental income opportunities through their sale in markets [5].

The cultural knowledge of wild food resources and the practices of hunting and gathering have been transmitted from generation to generation in rural Northeast Thailand. While it is important to recognize this knowledge, we must also recognize that knowledge, as a part of culture, is not static, nor does every member of a culture group hold the same amount of knowledge.

Under contemporary circumstances, rural villages in Northeast Thailand are experiencing a good deal of change. There is an increasing need for cash and much of the younger adult population has migrated to seek employment in Bangkok. Associated with this change is the concomitant change in household structure. The matrilocal residential stem family common to the Thai-Lao population in the region [17,23] is changing and household composition consists increasingly of grandparents raising grandchildren (primarily children of their daughters and son-in-laws). One of the primary questions we had in developing the research presented in this paper is what do children know about wild foods and is there a difference between children who are being raised by grandparents compared to those being raised by their parents? Will we find an indication of the hindrance of transmission that can lead to loss? This loss has been reported in a number of studies at various world locations [24-27].

Traditional ecological knowledge (TEK) or indigenous knowledge (IK) is largely transmitted through socialization within the household context. It requires family wholeness and considerable interpersonal relations as a channel of intergenerational cultural transmission and it is learned mostly along gender lines. Thus, the composition of the household may have significant implications for communication of knowledge and values to children [28,29].

This paper examines selected aspects of children's knowledge of wild food resources in a village in Northeast Thailand^1^. This paper also broaches the topic of children's acquisition of knowledge of wild food resources in their surroundings. The context of this study is one where the village has experienced change due to the migration of the parental generation where children are left behind in the village to be raised by their grandparents.

Attention has been paid to not only knowledge acquisition of children within the household, but also in the wider context of their interactions with the natural environment and social interactions with others within the community.

## Research area

**Figure 1:**
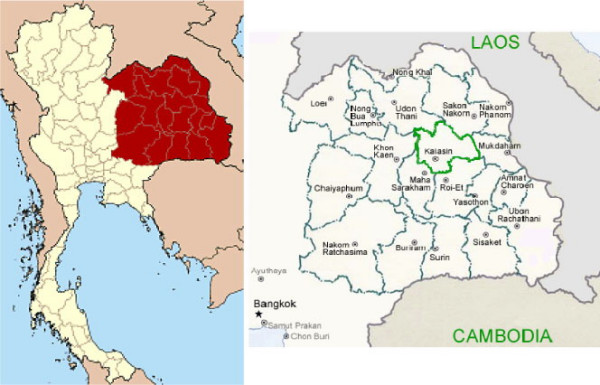
Map of research area. Kalasin province, Northeast, Thailand [45, 46].

The research was conducted in a small village in Kalasin province in the Northeast of Thailand. This region is known within Thailand as *Isaan *(Figure 1). The village is fairly typical of the region where hunting and gathering for wild foods is still practiced. The majority of people are rice farmers. Their core diet is glutinous rice with a wide variety of local wild and semi-domesticated plants and animals. These foods include a variety of weeds, birds, rats, lizards, snakes, insects, and frogs. They are found and collected in the surrounding areas such as roadsides, paddy field dikes, irrigation canals and swamps, depending on the season. [See Additional File 1 for the picture of boys and their snares, one of the tools used for catching rats in paddy fields.] Many of the households also grow a variety of domesticated food plants and fruit in their residential compounds and paddy fields and raise livestock such as cows, pigs and chickens.

Wild foods are considered by the rural population in the region as a necessity rather than as a supplement and are eaten daily in combination with rice [16]. Gathered wild foods represented more than 50 percent of the items that comprised the local diet during the rainy season [12], and gathering accounted for over 30 percent of food procurement activities during the dry season [8]. The study by Kunarattanapruk et al. found that 62 percent of household food was made up of wild food resources (31 percent came from the forest, and 31 percent came from the paddy field), 22 percent was produced by the household, 13 percent was purchased, and 3 percent were gifts [30].

Today rural northeasterners are increasing dependent upon a cash economy and market. Poverty, limited alternative sources of employment and a marginal environment characterize the region and are catalysts of migration to urban centers for wage labor. The household composition and other circumstances of the household have been significantly affected by migration. In the research village, there are 37 households with children age 0–15 years old among the total of 55 households. There are 22 households out of 37 with children whose parents are away due to migration. There are 60 children in this village (32 boys and 28 girls). 53.33 percent, or 32 children (18 boys and 13 girls), are living away from their mother and/or father due to migration. Mainly grandparents and sometimes other relatives are caretakers of these children while their parents are away, often years at a time. This phenomenon sets the context within which children in the present acquire their knowledge of food resources.

## Methods

The fieldwork was conducted in January and February 2007 during the cool/dry season. In the initial phase, the secondary data on household composition from the village census and the data of wild food plants were available to use as baseline data. These data were collected in the village for the research project "Wild vegetable, fruit and mushrooms in rural household well-being: An in-depth multidisciplinary village study in Northeast Thailand". Moreover, informal discussions and village walks with key informants, both adults and children, were held to enhance understanding and gather information about migration in the village and about different species of wild food plants and animals available around the village and the paddy fields. The free-listing was used to elicit the emic concepts and categories that belong in a domain of wild food of children. All 30 children (15 boys and 15 girls) aged 8–14 years old in the village from both migrant and non-migrant families participated in the free-listing. The ANTHROPAC program [31] was used to generate Smith's Salience Index. All above-mentioned information was useful for selecting informants and wild food species appropriated for further in-depth study.

The study in the later in-depth phase was primarily qualitative research using a case study approach in order to gain holistic in-depth insight into children's theoretical and practical knowledge, as well as patterns of how children acquire their IK regarding wild food resources.

There were 19 children (ten boys and nine girls) aged 10–12 years old within the village. Eight children (four boys and four girls) were purposefully selected as the key informants for an in-depth case study. Children aged 10–12 were selected because a number of studies on children's acquisition of traditional knowledge and skills show that children have already learned most of the tasks and skills and there will be minimal difference in the level of expertise of the children at these ages [24,28,32,33]. They were typical village children consisting of four children (two boys and two girls) from migrant families who live in the village away from their parents for at least six months a year, and another four children (also two boys and two girls) from non-migrant families. They and their families were willing to participate.

A standardized set of 20 species covering 10 species of edible plants and 10 species of edible animals were purposefully selected to be a main focus of the study in order to determine children's TEK of wild food plants and animals and how children acquire their practical knowledge. The species were selected according to a predetermined set of criteria and appropriateness for the research. These species were commonly found in the children's surrounding (within the village area, around their house, their school, and nearby cannels, swamps and paddy fields). The items ranged in different levels of saliency (results of the Smith's index) derived from all the village children's free-listing in the initial phase. The items chosen for more detailed study were accessible and safe for children to collect and they were available during the research period (season). The 20 species with scientific names are shown in table 1 (list of selected wild food plants) and Additional File 2 (list of selected wild food animals). The botanical names of plants were obtained from botanists working in the village. Scientific names of animals were obtained from biologists and a literature search.

**Table 1:** List of selected wild food plants

**Wild food plant name**	Smith's Salience Index*		**Gathering**	**Consuming**
			
	**Boy**	**Girl**		
1. Phak kaen khom () **Botanical name**: *Lobelia alsinoides *Lam. **Family**: Campanulaceae Common name: Chickweed	0.542	0.558	Gathered by hands from paddy fields and areas where there is moisture in the soil.	Most abundant and consumed in October to February after rice harvesting. It has bitter taste. Yod (tip of the plant that is new growth consisting of tender young leaves and stem) or whole young plant is eaten. Often cooked in curry.

2. Phak kayang () **Botanical name**: *Limnophila aromatica *(Lomk.) Merr. **Family**: Scrophulariaceae **Common name**: Rice Paddy Herb	0.230	0.217	Gathered by hands from paddy fields.	Available from May to November. Yod or whole young plant is eaten. Often cooked in curry.

3. Phak khee lek () **Botanical name**: *Cassia siamea *Lam. **Family**: Caesalpiniaceae **Common name**: Thai Copper Pod	0.167	0.259	Gathered by hands or fork stick from house area (transplanted), around the village, paddy fields, plantation areas, and secondary forests.	Most consumed in November to February. Yod and flower are eaten. Often cooked in curry.

4. Phak som/Phak kaen som () **Botanical name**: - **Family**: - **Common name**: -	0.169	0.135	Gathered by hands from paddy fields and areas where there is moisture in the soil. It looks similar to Phak kaen khom but has sour taste. These two species often grow together. Children have to learn to distinguish these two species when they collect.	Most abundant and consumed in October to March. Yod or whole young plant is eaten. Often put in curry to give some sour taste.

5. Phak mek () **Botanical name**: *Melaleuca quinquenervia *(Cav.) S.T.Blake **Family**: Myrtaceae **Common name**: Punk Tree/Paperbark Tea Trees	0.211	0.020	Gathered by hand from house area (transplanted), around the village, paddy fields, plantation areas and secondary forests.	Consumed all year round when new leaf buds emerge., especially during the rainy season. It has a slightly sour taste. Young leaf shoots are eaten raw.

6. Phak waen () **Botanical name**: *Marsilea crenata *C. Presl **Family**: Marsileaceae **Common name**: Water Clover, Clover Fern	0.156	0.073	Gathered by hand from house area (not transplanted), around the village, paddy fields and areas where there is moisture in the soil.	Most abundant and consumed in August to October. Young leaf shoot is eaten raw.

7. Phak hom () **Botanical name**: *Marsilea crenata *C. Presl **Family**: Marsileaceae **Common name**: Water Clover, Clover Fern	0.058	0.067	Gathered by hand from house area (not transplanted), around the village, and paddy fields.	Young plants are consumed all year round and especially during the rainy season. Young leaf shoot or whole young plant is eaten raw or parboiled.

8. Phak lin pii () **Botanical name**: *Emilia sonchifolia *(L.) DC. **Family**: Asteraceae **Common name**: Lilac Tasselflower	0.043	0.060	Gathered by hand from paddy fields.	Most abundant and consumed from November to December. Young leaf shoot and flowers are eaten raw.

9. Bak tong leeng () **Botanical name**: *Polyalthia evecta *(Pierre) Finet & Gangnep. **Family**: Annonaceae **Common name**:-	0.017	0.038	Gathered by hand from the village area, plantation area, and secondary forest.	Fruit is available from June to July. Fruit is eaten raw and often on spot as snack.

10. Phak lam () **Botanical name**: *Adenanthera pavonina *L. **Family**: Mimosaceae **Common name**: Red Beadtree	0	0.002	Gather by hand or long fork stick on pole from house area (transplanted), around the village, paddy fields, and secondary forests.	Different parts are eaten. Young leaf shoots and flowers are eaten fresh or parboiled. Seed/fruit is roasted and eaten as a snack. Raw seed/fruit is toxic.

* Smith Salience Index varies between 0–1. The most salient (basic) terms of the domain have the value 1. The term not mentioned at all (least salient) have the value 0.

One plant was not identified by scientific name. As local names of plants and animals can vary throughout the region, it should be noted that all the local Thai-Lao names of plants and animals used in this study are names commonly shared within the village.

Strategies used for data collection included participant observation, interviews, and photo identification. Participant observation in combination with informal interviewing was used in natural settings to enter informants' life worlds by integrating into children's activities. Child informants were accompanied particularly during their gathering activities as well as during their play. Practices and interactions the children had either with other children, their caretakers or others were noted. Caretakers as well as children were asked about the children's life histories. This technique captured the patterns of socialization and cultural knowledge transmission and practical dimension of the children's IK. Moreover, general socio-economic contexts of households and circumstances such as child rearing patterns, communication environment, consumption patterns, norms, values and the culture of gathering and consuming wild foods of each child and his or her family were observed and recorded.

Photo identification exercises and semi-structured interviews were conducted with each child. Children were asked to identify the photos of 20 species. The interviews were guided by a list of open-ended questions and were conducted to measure the practical knowledge of children about these plants and animals. To measure children's knowledge, scores were given based on the correct answers. The answer key was derived from secondary data (generated in this village in 2005 and 2006), discussions, and participant observation with key informants (both adults and children). Each species has the full score of five, which consists of one point for correctly naming the item (photo identification), one point for correctly identifying at least one edible part, one point for correctly identifying the gathering season, one point for correctly identifying at least one gathering location (environment) and one point for correctly identifying at least one technique in gathering. As 20 species were studied, the potential full score for each child would be 100.

The knowledge scores and in-depth detailed data derived from participant observation and interviews are juxtaposed and analyzed in order to understand children's knowledge and the nature of knowledge transmission in this specific context.

## Results

### Children's traditional ecological knowledge of wild food resources

From the free-listing with 30 children, a total of 77 wild food plants and 86 wild food animals were mentioned. Out of 77 wild food plants, 7 were aquatic plants; 11 were herbaceous; 38 were trees; 10 were vines; 1 was a bamboo species; 2 were palms; 4 were shrubs; 2 were rhizomes; and 2 were tubers. Most plants have multiple uses and different parts of plants are eaten. This inventory included both fruit and vegetables. Names of fruit were frequently mentioned among children. This seems logical because most of the children like fruit and they generally collect these in a group and share them with friends as snacks when they go out and play together. [See Additional File 3 for the picture of fruit gathering.] These fruits are *Bak kham *(*Tamarindus indica *L.)*, Bak muang *(*Mangifera indica *L.)*, Bak yom *(*Phyllanthus acidus *(L.) Skeels), *Bak than (Ziziphus mauritiana *L.), *and Bak tong leeng (Polyalthia evecta *(Pierre) Finet & Gangnep.). The vegetables *Phak bung *(*Ipomoea aquatica *Forssk.*), Phak kaen khom *(*Lobelia alsinoides *Lam.)*, Phak kasek (Leucaena leucocephala *(Lam.) de Wit.),*Phak tamnin *(*Coccinia grandis *(L.) Voigt),*and Phak kayang *(*Limnophila aromatica *(Lomk.) Merr.) have the highest saliency scores, meaning that they were mentioned frequently and early in the free listing exercise by children. Based on interviews, these species are easily found in the surroundings, are often consumed, and considered by many children to be their favorite wild food plants, and have high market value.

In a previous study, Price elicited 77 wild food plants from adult women in the village [8]. Although there are differences in content, the number of plant species elicited from children in this study was as many as the species elicited from adult women in Price's earlier study. This shows the ability and familiarity of children regarding the naming wild food resources.

Out of 86 wild food animals, the children named 22 types of fish, 20 types of insects (found on land or water), and 19 types of birds. It is noteworthy that names of fish were often mentioned by both boys and girls. In the Northeast, fish is very important and it plays a major role as a protein food in the diet. Moreover, there are also canals and swamps around the village where villagers can acquire fish for their meals. All sorts of fried insects are considered a delicacy for children and they like to eat them as part of their meal as well as consuming them as snacks. These insects are good sources of protein for children at a lower cost. All 19 types of birds were only mentioned by boys. This might be because birds are in general caught only by men and boys. Eight types of amphibians including tadpoles and different kinds of frogs, seven types of reptiles such as lizards, snakes and turtles were mentioned. Seven types of shellfish were listed, including different kinds of snails, mussels, crabs and freshwater shrimp. The three mammals mentioned are all different kinds of rats found in the paddy fields. The most salient wild animal foods for the children are the fish *Pla kho *(*Channa striata)*, the rat *Nuu *(*Ruttus rattus)*, the fish *Pla kheng *(*Anabas testudineus)*, the fish *Pla duk *(*Clarias batrachus*), and the freshwater paddy field crab *Puu na *(*Somanniathelphusa *sp.).

### Gender differences in children's TEK

Girls free listed 49 plants and 45 animals. Boys free listed 67 plants and 81 animals. Boys could give more names than girls and they could name animals more than plants. This may be because boys spend more time wandering around the village and playing outside with friends and at the same time collect and consume wild edibles such as fruit on the spot.

Twenty species of wild foods were selected for in-depth study in order capture the naming ability (theoretical knowledge) and practical knowledge in acquiring these wild resources among children in the village. These species are shown in Table 1 (list of selected wild food plants) and Additional File 2 (list of selected wild food animals). Table 2 displays information about the in-depth study informants, their age, household composition and their knowledge scores for both wild food plants and animals.

**Table 2:** Information of children key informants and their knowledge scores

Children key informants	Age	Household composition		Score	
			
			Plants	Animals	Total
**Boys**					
Wora	12	Grandmother and younger brother (grandfather is deceased, parents are absent due to migration)	29/50	43/50	72/100
Wancha	12	Grandmother, grandfather, and younger brother (parents are absent due to migration)	35/50	50/50	85/100
Panu	11	Mother, father, and elder brother (grandmother lives in the same compound)	30/50	50/50	80/100
Weepat	12	Mother, father, and elder sister (grandmother lives in the same compound)	24/50	45/50	69/100

Total			118/200 (59%)	188/200 (94%)	306/400 (76.5%)

**Girls**					
Porn	11	Grandmother and grandfather (parents are absent due to migration)	25/50	33/50	58/100
Nipa	10	Grandmother, aunt, and a 12 year-old femalel cousin who is the daughter of the aunt (parents are absent due to migration, grandfather is absent due to death)	39/50	43/50	82/100
Arinee	11	Grandmother, grandfather, mother, father, and younger brother	50/50	49/50	99/100
Sarin	10	Mother, father, and younger sister (grandmother lives in the same compound)	19/50	38/50	57/100

Total			133/200 (66.5%)	163/200 (81.5%)	296/400 (74%)

Using the knowledge scores, we conducted the Mann-Whitney Rank-sum Test^2 ^on boy's and girl's scores. The results of this test indicate that there is no statistically significant difference in the knowledge scores of boys and girls on plants and animals, nor in the overall knowledge score. It should be noted that the small sample size may be an important factor in the above outcome. The in-depth results, however, do show a difference even if not statically significant. Girls have a higher level of knowledge about plants (66.5 percent) compared to boys (59 percent), while boys have a higher knowledge about animals (94 percent) compared to girls (81.5 percent). This gender difference, related to the sexual division of labor in gathering and hunting of wild food resources, and gender differences of male and female skill, was also reported in other studies conducted in the northeast [34,35]. Women and girls predominate in gathering wild food plants. For animals, some species are obtained by both genders, while others are mainly gathered/captured by men or women. The results of the present study show that girls have higher skill knowledge about plants but less skill knowledge in procuring fish, paddy rats, water insects and tree lizards. Girls reported that they also like and consume these animals as well, but boys are more active in gathering and hunting these species.

### Children's valuation of wild food resources

Value significantly links to practices and the TEK learning process. Children's practical knowledge about each species depends on their own valuation in relation to characteristics of specific wild food resources, but their values are also part of the larger context of valuation in their homes and communities. Children have better knowledge about plant and animal species that they have hands-on experience in gathering either for sale or consumption. These species are regarded as having desirable taste (including texture and smell for plants) and high marketability (see [8] on adult women's evaluations). Taste and marketability are primarily factors that influence children's valuation and, as a result, their gathering and/or consuming activities. These valuations and practices in the end are important variables that affect the level of their practical knowledge.

**Figure 2:**
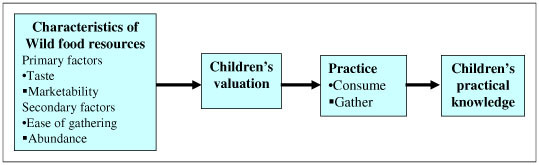
Simplified diagram shows association between children's valuation and their practical knowledge.

Ease of gathering and availability/abundance in their surroundings are supplemental to these primary factors. Children have the most practical knowledge about species which they consider to have a desirable taste and high marketability, as well as being easy to gather and abundant in the surroundings. However, children have less knowledge about certain plants even though they can be easily found and gathered around the house and paddy fields when they have less desirable taste and lower marketability. Figure 2 shows a diagram illustrating the association between children's valuation and their practical knowledge.

This emphasis on the interlinking qualities of wild food plant taste, marketability and abundance (or rarity) have been absorbed by the children in this study from the larger community. These qualities have been shown to mark cultural significance as well as culturally prescribed gathering rights and restrictions in this same village as documented in the literature [8,11,17]. Thus, not only are children leaning about these foods, but they are learning about food plants in a very species-specific manner that is culturally determined through women. One difference, however, is that the rarer a species is for adult women (with high market and taste value), the more restricted the gathering rights for these plants become on the property of other people. For the children, abundance and ease of collection emerged as important factors in actual acquisition of food items. There are generally no community prohibitions on gathering from other people's property if the plant species is considered abundant [8,11,17].

There are species in this study, especially wild food plants, which children have less knowledge about. These plants are *Phak waen, Phak hom *and *Phak lin pii*. Children reported that they recognize the name of these plants as they have heard them mentioned but they themselves have never or rarely gathered or eaten them. The children who know and have ever eaten them said they do not like to eat them much as they do not taste good, they are bitter, tasteless or have a strong smell. On the contrary, children have good knowledge about *Phak kaen khom *and some wild food animals such as paddy rats, paddy crabs and snails as they sell well at the market in the provincial capital and are easily found around the village and paddy fields. Children see their family members and other people in the village gather these species in the fields and they engage in gathering them themselves.

### Knowledge acquisition of children in the village context

Children gain traditional knowledge of wild food resources through their lived experience. The specific local context of culture, society, economy, and bio-physical-environment are important elements in the kind of knowledge children gain. Equally important to traditional knowledge acquisition are the interactions with family members and other members of the community (knowledge holders). TEK learning processes consist of observation, participation and practice. The different aspects of TEK (e.g. theoretical, practical, belief, value) are generated and transmitted during these interactive processes. The process of knowledge acquisition can take place both across and within generations. The level of children's knowledge varies depending on a large number of interrelated factors. This section explores the influence of social interactions on children's indigenous knowledge.

A number of studies have confirmed that traditional knowledge is largely transmitted vertically and parents are the main contributors in knowledge transmission [24,28,29,36]. Women, especially the mother and grandmother, are often regarded as the primary transmitters of wild plant knowledge [36,37]. While parental transmission is undoubtedly important, other knowledge acquisition channels are not negligible. Especially in the context where the parental generation is absent or mostly absent, the contribution of other social contacts and interaction channels within the context of the natural environment and the community are considerable.

Grandparents are the main caretakers for children in the absence of the parental generation in the research village. Children spend a large portion of their time with grandparents and friends. Children reported that they learned about wild food resources from grandparents, their brothers/sisters, cousins, village friends, other adult relatives and neighbours when their parents are not around. Children who acquire knowledge thorough an across-generation transmission (parents, grandparents, other adult relatives) can learn from skilful adults, receiving proper guidance and supervision. Grandmothers and mothers contribute to teaching children about wild food plants and some animals which are mostly gathered by women. Grandfathers and fathers teach techniques in acquiring wild food animals which are hunted and collected by men.

Children generally started to learn about gathering and hunting wild food resources at the age of 7 when taken into the field and joining in the collecting trips with adults. At the age of 10–12, children have gained a large part of their knowledge. At the same time, grandparents became less active in gathering and hunting activities as they grow older and some also have to take care of other younger grandchildren in the family. They let the older children wander around on their own, with friends and cousins mainly of the same age and sex.

There are also children in the village who primarily acquire their knowledge of wild food resources through peers of the same generation as their parents are away and they have not engaged in gathering with their grandparents. Children who learned less from their caretakers had an opportunity to learn the practical knowledge about wild food resources with friends during their play and interactions together in the fields. During collecting trips with other children, knowledge is shared and learned among them. For children especially at the ages of 10–12, the peer group is observed to be a crucial channel of knowledge acquisition. Gathering and hunting activities are often combined with play and wandering around in the surroundings in bands with other children. Rogoff and Cruz also found this pattern of children's interactions in their research [37,38]. Rogoff suggests that direct interaction of Kenyan children with adults declines significantly by age as they engage more with other children in the same age and same sex cohort [38]. Cruz has observed that many children collect wild plant foods in groups together with friends and consume these on the spot [37]. These activities provide opportunities for children to share their knowledge with friends. Therefore, peers are viewed as another important avenue in the communication and exchange of local knowledge.

Apart from combining gathering and hunting with other activities such as play, children also make excursions specifically aimed at gathering and/or hunting. In this case, the division along gender lines is clear. Their companions are mostly their siblings, cousins, and neighbor children of the same sex. Moreover, children at this age start to have chores within a household. Their collecting activities might also be conducted alone while engaging in the chore of taking care of the family's cattle in the field, when the child may be asked to go out and collect wild foods for the family's meal.

Skill and mastery depend, of course, on a large number of factors, but mainly the frequency of practice and the amount of contact with transmitters within the specific context. Some children, even though living in the household together with both grandmother and grandfather, do not engage often in gathering with them and at the same time are isolated from friends. These children do not learn as much about wild food resources in their surroundings. They may learn the name of the plants and animals as they have heard the name, eaten the item or seen it out of context, but do not know how, when and where to gather them.

## Discussion and conclusion

This study presents an exploratory look at children's theoretical and practical knowledge of wild foods in a village in Northeast Thailand. The study further examines the link this knowledge has with species valuation (plants) and the process of knowledge acquisition in a context where migration of the parental generation is prominent.

As the results of this study on children's knowledge show, there is no significant difference among children who are being raised by grandparents and those being raised by their parents. There are some explanations we propose for this. We have observed that there are different channels of knowledge transmission to be taken into consideration. Boys and girls learn their practical knowledge of wild food acquisition from grandparents, peers, and others in the community, including close relatives and neighbors.

However, the process of TEK learning is more complicated. The theoretical dimension of TEK in this study refers to children's ability to name wild food resources. This knowledge is acquired when children live in the specific local context. They see these natural foods, they eat them, and they hear others talk about them, so they learn what they are called. Another element of TEK is a technical element, which is practical skill knowledge and mastery. This element is even more crucial for local people in that they apply it to acquire their foods from nature. The practical knowledge requires a higher degree of involvement in order to learn. It is the knowledge that provides detail and specific information about these resources such as where, when, and how to acquire and prepare them. Some species might be poisonous or require specific techniques in gathering or processing. Therefore, it is this part of knowledge that helps village people to fully receive the benefits from the natural food resources and enhance their food and livelihood security.

In this study, the theoretical knowledge of the village children in general remains quite high, as evident in their ability to name wild food plants and animals in the free-listing task. This is particularly prominent in the case of wild foods, where boys could name many species and more than girls in the free listing exercise. For plants, boys free listed 67 items and girls 49. This is also the case with animal naming, where boys listed 81 items and girls only 45. Further, boys listed 19 types of birds and girls none.

However, the children's practical knowledge and mastery in procuring these wild food plant resources were lower than anticipated, as many children had difficulties identifying and answering questions about how to acquire these wild species. Boys scored in their answers to practical questions at only 59 percent correct, with girls having a score of 66 percent. Girls had more practical knowledge and mastery compared to boys with regard to wild food plants. With regard to animals, boys showed mastery at a level of 94 percent and girls at 81.5 percent. Adults expressed their surprise during the children's interviews when their children did not know the answers with regard to plant foods. Some adults had not realized that their children had such limited practical information about these wild food plants.

TEK is a tacit or an implicit type of knowledge that people know and apply but do not normally express. Moreover, it is localized because it gains a particular place only through experience and practice by a particular community environment. It is also not static, but dynamic as it expands or reduces according to the changes in the context. In addition, the way knowledge is transmitted is not institutionalized like scientific knowledge that is widely taught in schools. Transmission of TEK is thus demanding and requires engaging in activities. This makes this type of knowledge vulnerable to decrease, especially in the face of environmental and societal changes within the village.

It was apparent during the field work that the transmission of TEK both across and within generations play roles in concert. In the early stage, adults play an important role in being examples and introducing and teaching children their food ways. Later when children become more involved with friends, they exchange and expand their knowledge through play and experimentation in the field together. Children who did not have a chance to learn a substantial part of the knowledge from adults can gain knowledge while interacting and communicating with their peers and other villagers during this phase. The data obtained in this study shows that the village children are still familiar with their wild food resources and these foods remain in their diet. At the same time, there is an indication that some species, especially wild food plants, are not clearly in their knowledge domain. Children have only heard the name of numerous species but never collected or consumed them. We are of the opinion that further research is needed in order to fully comprehend the factors that contribute to this knowledge gap. It is possible that the increased rarity of selected species in the natural environment can be a contributing factor. Likewise, the growing availability of processed market foods accompanied by newly acquired tastes by the children may also be a contributing factor. Gathering and consuming less may ultimately bring about a further erosion of knowledge for some species in the future.

## Appendix

^1 ^The project "Wild vegetable, fruit and mushrooms in rural household well-being: An in-depth multidisciplinary village study in Northeast Thailand" is a collaboration between four universities and the baseline data used in this study was collected in 2005/2006 by Dr. Viyouth Chamruspanth of Khon Kaen University (village census); Dr. Lisa Leimar Price of Wageningen University (local names, environments, gathering seasons and rights), and botanical identification coordinated by Dr. Chayan Picheansoonthon of Khon Kaen University. This project is under the support of the Neys-van Hoogstraten Foundation.

^2 ^The Mann-Whitney Rank-sum Test (U Test) is appropriate for non-parametric data which accomplishes essentially what at test does [39].

^3 ^Botanical and zoological scientific names in this study have been identified by botanists and biologists (consisting of Dr.Chusie Trisonthi, Dr. Saismorn Lumyong, Dr. Chayan Picheansoonthon, Ms. Pornpimon Wongsuwan, and Mr. Jose Coltro) using fresh specimens. Other reference materials utilized for identification include [40-43] Kasetsart University Agricultural Information [44], the International Plant Names Index (IPNI), and ZooBank prototype: the world register of animal names based on Thomson Zoological's "Index of Organism Names."

## Abbreviations

TEK Traditional Ecological Knowledge

IK Indigenous Knowledge

## Competing interests

The author(s) declare that they have no competing interests.

## Authors' contributions

Both authors share the contribution to compilation of this manuscript.

## Supplementary Material

Additional file 1 Rat catching. Boys were putting snares in the paddy fields to catch rats in the evening. Click here for file

Additional file 2 List of selected wild food animals. Table showing names and detailed information of selected wild food animals in this study. Click here for file

Additional file 3 Fruit gathering. Children were climbing up the tree to gather fruits. Click here for file
