# Supplementary figures and images for: Children's traditional ecological knowledge of wild food resources: a case study in a rural village in Northeast Thailand
Source: J Ethnobiol Ethnomed. 2007 Oct 15;3:33. doi: 10.1186/1746-4269-3-33 (PMC2100045; doi:10.1186/1746-4269-3-33)

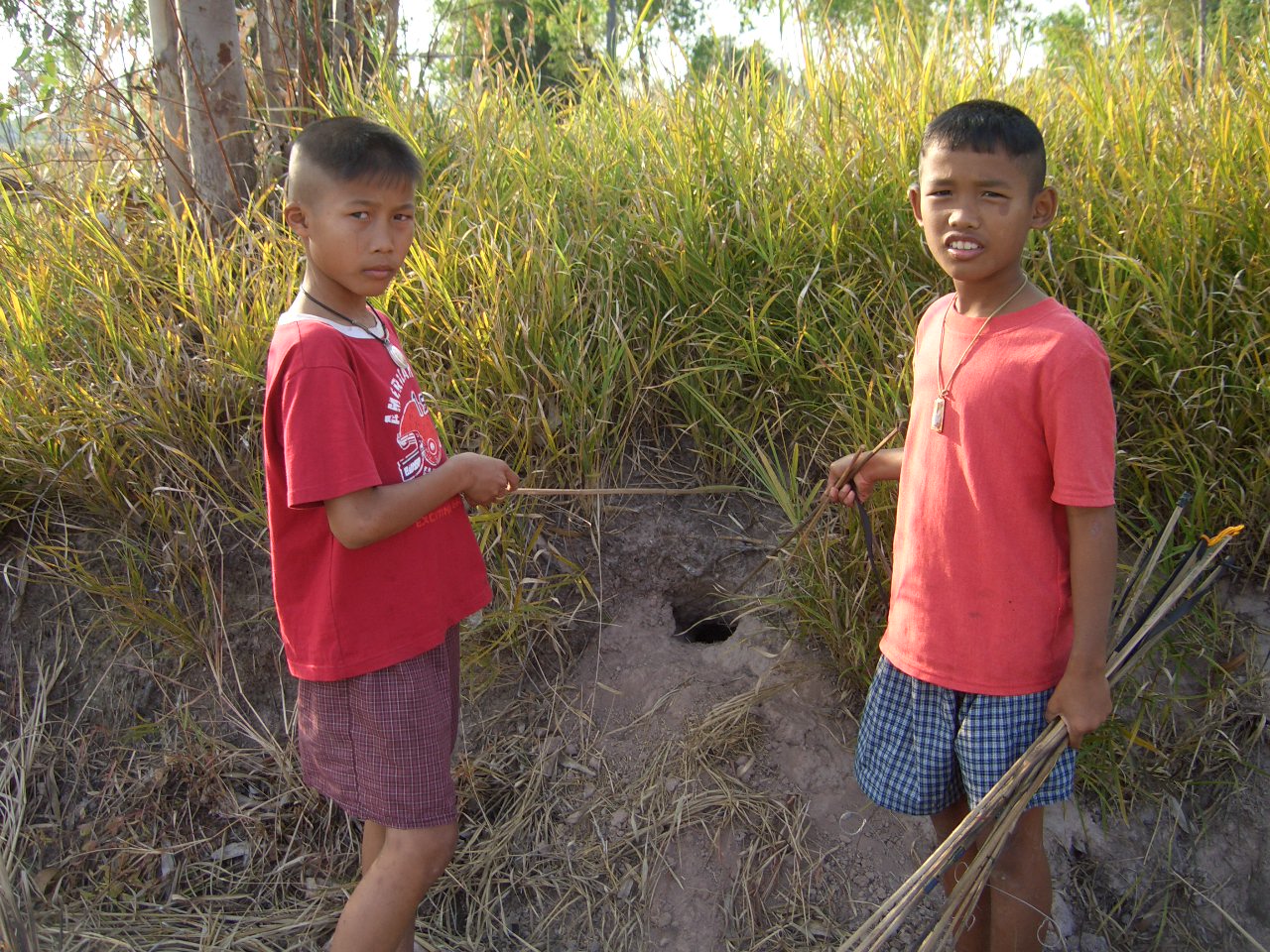

Supplement: Additional file 1 — Rat catching. Boys were putting snares in the paddy fields to catch rats in the evening. [file 1746-4269-3-33-S1.jpeg]

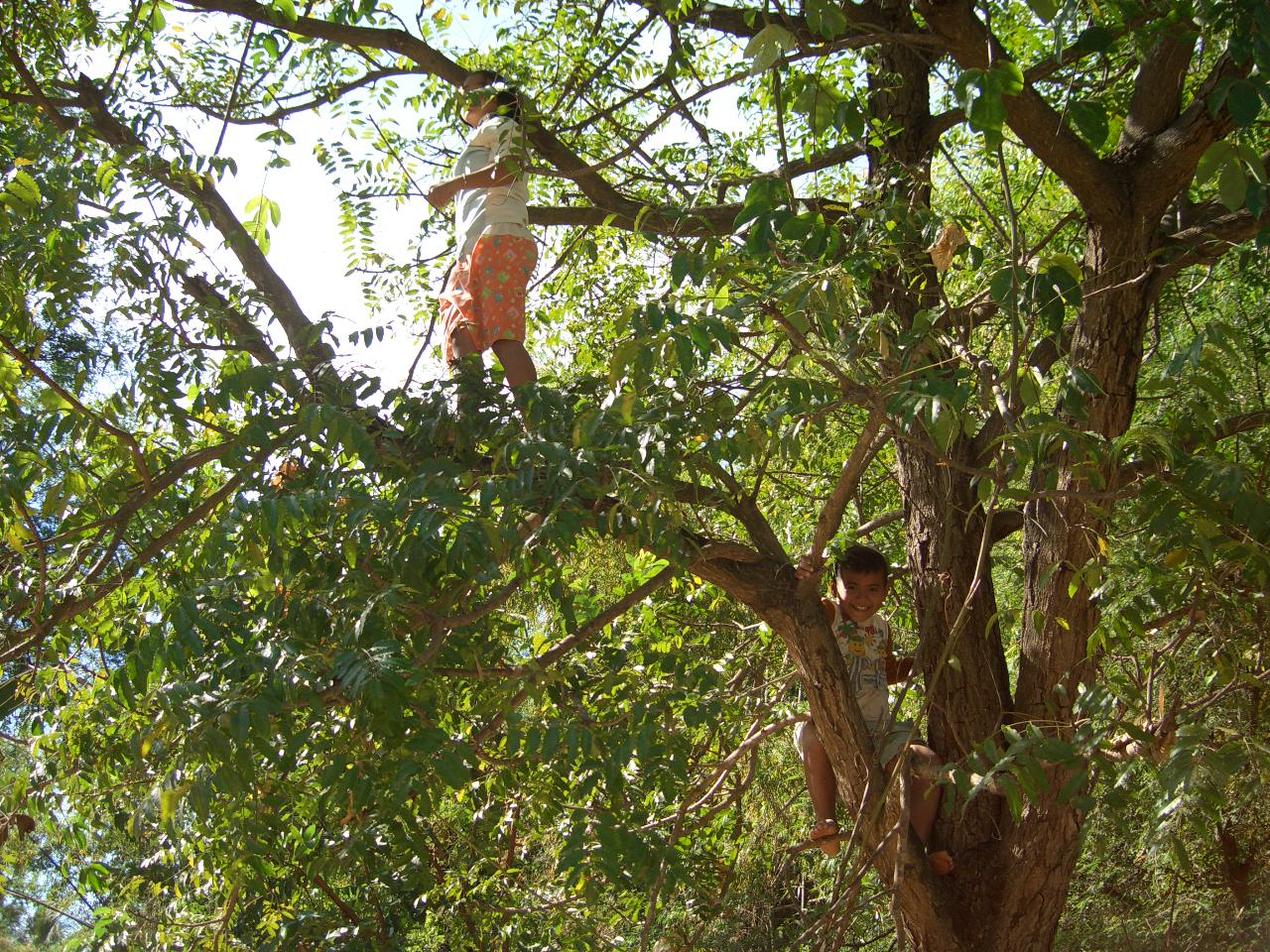

Supplement: Additional file 3 — Fruit gathering. Children were climbing up the tree to gather fruits. [file 1746-4269-3-33-S3.jpeg]
