# Supplementary material for: Children's traditional ecological knowledge of wild food resources: a case study in a rural village in Northeast Thailand
Source: J Ethnobiol Ethnomed. 2007 Oct 15;3:33. doi: 10.1186/1746-4269-3-33 (PMC2100045; doi:10.1186/1746-4269-3-33)
Supplement: Additional file 2 — List of selected wild food animals. Table showing names and detailed information of selected wild food animals in this study. [file 1746-4269-3-33-S2.pdf]

List of selected wild food animals

Wild food animal name	Smith's Salience Index*		Gathering	Consuming
	Boy	Girl		
1. Nuu (หนู)<br>**Scientific name**: *Ruttus rattus*<br>**Common name**: Paddy rat	0.879	0.195	Caught by different techniques. Use smoke to chase them out from their holes and catch by using slingshots or hands. Imitate their squeak or use rice to lure them from their hole. Catching rats is done at dusk or at night. Many times traps are put out along the rat's routes around the paddy fields in the evening and rats be collect early the next morning. Snares are made from bamboo sticks with a metal or rubber loop. Rats are mainly caught by men and boys.	Grilled, cooked in curry or fried. Rat is considered a favorite food by many children.
2. Puu na (ปูนา)<br>**Scientific name:** *Somanniathelphusa* sp.<br>**Common name:** Paddy crab	0.275	0.494	Collected by hand or with fishnet together with fish from canals, swamps, ponds and flooded rice paddy fields during the rainy season. During the dry season, crabs live under dried mud. Collectors use a spade to dig in their holes and catch them. Generally collected by women and children.	Grilled with salt, cooked in curry, or fermented in salt. The meat is eaten together with some soft parts of the shell.
3. Kapom (กะปอม)<br>**Scientific name:** *Calotes versicolor*<br>**Common name:** Crested Tree Lizard	0.546	0.179	They are caught around the village, house areas, plantation areas, secondary forest and paddy fields. They use a slingshot for shooting or bamboo stick with a loop at the tip of the stick to hook the lizard's neck. Lizards are mainly collected by men and boys.	They are mostly caught during the dry season as villagers said the meat is most delicious then. They are flayed and grilled.

4. Pla kayang (ปลากะแยง/ปลาแขยง)<br>**Scientific name:** *Calotes versicolor*<br>**Common name:** Crested Tree Lizard	0.162	0.117	They are usually caught from canals by using a fishnet. They are mainly caught be men.	Grilled with salt, cooked in curry, or fermented in salt.
5. Hoy Sherry (หอยเชอรี่)<br>**Scientific name:** *Pomacea canaliculata*<br>**Common name:** Golden apple snail	0.142	0.078	Collected by hand or with a handnet from canals, swamps, ponds and flooded rice paddy fields during the rainy season. During the dry season, snails live under dried mud. Collectors use a spade to scrape the ground to find and catch them. Generally collected by women and children.	Snails are cleaned and parboiled. They are taken out from their shells, cut, and cleaned in salted water. After rinse with water, they are mixed with roasted rice, dried chili, lime juice, and fish sauce.
6. Huak (ฮวก)<br>**Scientific name:** *Rana* spp.<br>**Common name:** Tadpole	0.031	0.140	Collected by using a handnet from canals, swamps, and flooded paddy fields during May and June. Women and children are primary collectors.	Put in curry or mixed with spices and herbs, wrapped in banana leaves and grilled (Mok).
7. Kead noi (เขียดน้อย)<br>**Scientific name:** -<br>**Common name:** Small floating frog	0.045	0.114	Small juvenile frogs of various species. Collected by hands from swamp areas, canals, paddy fields and secondary forest. They are also collected by using spade to graze into the ground to find and catch them. Collected by both men and women.	Cooked in curry or other local dish called Mok.
8. Kung foy (กุ้งฝอย)<br>**Scientific name:** *Macrobrachium lanchesteri*<br>**Common name:** Small freshwater shrimp	0.041	0.110	Collected by using fishnet or handnet from canals, swamps, and flooded paddy fields. Mainly collected by women.	Shrimps are eaten cooked or raw in a local dish. They are also fermented together with small fish.

9. Hoy joob (หอยจูบ)<br>**Scientific name:** *Viviparus quadratus disparis*<br>**Common name:** Pond snail	0.116	0.024	Collected by hand or with handnet from canals, swamps, ponds and flooded rice paddy fields during the rainy season. During the dry season, snails live under dried mud. Collectors use a spade to scrape the ground to find and catch them. Collected by both men and women	Snails are cleaned and put in curry. They are also parboiled in salted water and eat together with green papaya salad.
10. Mang na ngum (แมงหน้างํ้า)<br>**Scientific name:** *Crocothemis* sp. (*Odonata, Libellulidae* spp.)<br>**Common name:** Common skimmer, nymph	0	0	Collected by using fishnet or handnet from canals, swamps, and flooded paddy fields. Mainly collected by women.	Most consumed during May and June Put in curry or other local dishes.

* Smith Salience Index varies between 0-1. The most salient (basic) terms of the domain have the value 1. The term not mentioned at all (least salient) have the value 0.
